# Virophages Found in Viromes from Lake Baikal

**DOI:** 10.3390/biom13121773

**Published:** 2023-12-11

**Authors:** Sergey Anatoljevich Potapov, Olga Ivanovna Belykh

**Affiliations:** Limnological Institute Siberian Branch of the Russian Academy of Sciences, Ulan-Batorskaya 3, Irkutsk 664033, Russia; belykh@lin.irk.ru

**Keywords:** Lake Baikal, virophages, high-throughput sequencing, biodiversity, viruses

## Abstract

In this study, a previously little-studied group of viruses—virophages—was searched for and identified in the viromes of the ancient oligotrophic Lake Baikal. Virophages are small dsDNA viruses that parasitize giant viruses (e.g., *Mimiviridae*), which in turn affect unicellular eukaryotes. We analyzed eight viromes obtained from the deep-water areas of three basins of Lake Baikal and the shallow-water strait Maloye More in different seasons. The sequences of virophages were revealed in all viromes and were dominant after bacteriophages and algal viruses. Sixteen putative complete genomes of virophages were assembled, all of which contained four conserved genes encoding major capsid protein (MCP), minor capsid protein (mCP), maturation cysteine protease (PRO), and FtsK-HerA family DNA-packaging ATPase (ATPase). The MCP-based cluster analysis showed a sequence separation according to seasons, and a dependence on the geographical localization was not detected.

## 1. Introduction

In 2008, *Acanthamoeba polyphaga* mimivirus (APMV) was isolated by inoculating *A. polyphaga* with water from a cooling tower. Using transmission electron microscopy, in addition to the giant virus APMV, a small virus with icosahedral virions 50 nm in size was observed, the genome of which was sequenced and found to be 18 kbp in length. The authors first proposed the term “virophage” in analogy to bacteriophage and named it Sputnik [1].

Subsequently, other virophages were discovered, including Mavirus [2], Sputnik strain Rio Negro [3], Sputnik2 [4], Sputnik3 [5], Zamilon [6], Zamilon2 [7], and many others [8,9]. The wide geographical distribution of virophages and their occurrence in different habitats are confirmed by their detection in metagenomes from Antarctica [10,11], a freshwater lake (China) [12], a Tibetan mountain lake [13], Yellowstone Lake [11,14], rumen samples from sheep [15], Lake Mendota and Trout Bog Lake [16], as well as from seawater, rivers, wastewater, and sediments [11]. In addition, the Chlorella virus virophage SW01, related to the large Chlorella virus XW01 infecting the unicellular green algae *Chlorella* sp. [17], was recently identified. To date, only 22 virophages have been formally characterized, i.e., for which a giant host virus and/or a eukaryotic host has been described; the rest have been identified in metagenomic assemblies [18].

Virophages are proposed to be related to the family *Lavidaviridae* with two genera *Sputnikvirus* (Sputnik and Zamilon) and Mavirus, based on six conservative proteins: major capsid protein (MCP), minor capsid protein (mCP), also known as penton, FtsK-HerA family DNA-packaging ATPase (ATPase), maturation cysteine protease (PRO), primase-superfamily 3 helicase (S3H), and a zinc-ribbon domain protein [19]. Later, only four of them were found to be “core” and two (S3H and the zinc-ribbon domain protein) were reclassified as “near-core” [16]. Next, 328 new virophage genomes containing all four main genes, MCP, mCP, ATPase, and PRO, were identified in 14,000 different publicly available microbiomes. Based on the data obtained, the classification of the family *Lavidaviridae* was revised [20]. 

Recently, S. Roux et al. proposed a new classification of virophages based on the combination of comparative genomic and phylogenetic analyses. The study suggests a division into four orders and seven families: (1) *Divpevirales*: *Ruviroviridae,* (2) *Lavidavirales*: *Maviroviridae*, (3) *Mividavirales*: *Sputniviroviridae*, and (4) *Priklausovirales*: *Dishuiviroviridae, Omnilimnoviroviridae, Gulliviroviridae, and Burtonviroviridae* [21].

As mentioned above, virophages are found in various ecosystems and associated with viruses related to *Mimiviridae* or viruses infecting phytoplankton. The eukaryotic host of giant viruses has been shown to be algae and protists [22]. Some authors hypothesize that virophages play an important ecological role in regulating the abundance of giant viruses, thereby increasing the survival rates of eukaryotic hosts [1,2,10]. For example, it is predicted that virophages in an Antarctic lake stimulate algal production by reducing overall mortality and thereby increasing the frequency of blooms [10]. 

It has been shown that the virophages Sputnik, Sputnik 2, and Sputnik 3 are able to infect viruses of the family *Mimiviridae* of three phylogenetic lineages (A, B, and C) [5], whereas the virophage Zamilon has a narrower host range and can only infect groups B and C [6]. The family *Mimiviridae* consists of three subfamilies: *Megamimivirinae* (representatives infect the Amoebozoa amoeba), *Klosneuvirinae* (infect amoeba and kinetoplastid phagotrophic protozoan), and *Aliimimivirinae* (infect bicosoecid phagotrophic protozoan) (https://ictv.global/taxonomy/taxondetails?taxnode_id=202203885, date of access 18 August 2023). Previously, the mimiviruses were divided into three lineages based on the *Pol B* gene sequences [5,23] and correspond to group A (including Mimivirus and Mamavirus), group B (Moumouvirus) [24], and group C (Megavirus chiliensis) [25]. Representatives of the family *Phycodnaviridae* also act as hosts for virophages, such as the *Phaeocystis Globosa* Virus Virophage (PGVV), which is derived from the giant virus *Phaeocystis globosa virus* (PgV–16T) that infects algae of the genus *Phaeocystis* [26]. 

In 2015, G. Blanc and colleagues discovered provirophages by identifying about 300 putative genes of virophage origin in the nuclear genome of the unicellular alga *Bigelowiella natans* [27]. The authors hypothesized that the integration of virophages into the genome of *B. natans* could be beneficial for both, as it leads to the protection of the latter from infection and the virophages benefit from an increased chance of encountering the giant virus. Virophage integration is also found in *Acanthamoeba polyphaga* (Lentille virus) [4] and in the self-synthesizing mobile element of *Maverick/Polinton* [2].

The ability of Mavirus to insert itself into the genome of the cultured protist *Cafeteria burkhardae* was previously tested. Eight different types of endogenous virophages (endogenous mavirus-like elements, EMALE) were discovered based on dot plots and phylogenetic analyses related to maviruses. EMALE can potentially re-activate and replicate in the presence of giant viruses [28].

We provide brief and basic information on virophages here because recent review articles fully reflect the current state of research on virophages [18,22,29,30,31]. To date, there is no detailed information on the presence of virophages in the ancient oligotrophic Lake Baikal, just as there are no data on virophages in other ancient and large lakes of the Earth. Previously, we and our colleagues conducted a detailed study of the DNA- and RNA-containing viral communities of Lake Baikal using amplicon and high-throughput metagenomic sequencing [32,33,34,35], thereby showing the diversity of viral communities.

The aim of this study was to identify virophages in the metagenomes of the viral fraction (smaller than 0.2 μm) from Lake Baikal using bioinformatic methods.

## 2. Materials and Methods

### 2.1. Sampling Sites

In 2018, water samples were taken 7 km from the settlement of Listvyanka (BVP1), 3 km from the settlement of Listvyanka (BVP2), 3 km from the settlement of Turka (BVP3), 3 km from Elokhin Cape (BVP4), at the central station in Maloye More Strait (BVP5), at the central station of the transect between the settlement of Listvyanka and the settlement of Tankhoy (BVP6), at the central station of Ukhan Cape—Tonky Cape (BVP7), and at the central station of Elokhin Cape—the settlement of Davsha (BVP8). The dates and coordinates of the sampling can be found in Table 1.

Sampling was carried out on board the research vessels of the Limnological Institute Siberian Branch of the Russian Academy of Sciences (LIN SB RAS) using the SBE-3 bathometer system (Carousel Water Sampler, Sea Bird Electronics Inc., Bellevue, WA, USA). From each horizon (0, 5, 10, 15, 20, 25, 50 m), 3.5 L was sampled and mixed to obtain an integral sample of the 0–50 m layer (a total of ~25 L for each sample). During the ice-cover period, sample BVP1 was collected under ice using Niskin bottles.

The samples were then filtered through 0.4 µm and 0.2 µm pore diameter polycarbonate filters (Millipore, Burlington, MA, USA) to remove phyto-, zoo-, and bacterioplankton. The filtrates were concentrated to a final volume of ~20 mL using a VivaFlow 200 tangential flow ultrafiltration system (Sartorius, Gottingen, Germany). Further, the specimens were concentrated to a volume of ~100 µL using Vivaspin Turbo 15 (50 kDa) (Sartorius, Gottingen, Germany).

To obtain free virus particles, the sample was treated with DNase (Thermo Fisher Scientific, Waltham, MA, USA) at 37 °C for 30 min. The DNAase was deactivated by adding 20 µL of EDTA 50 mM at 65 °C, which was held for 10 min. The presence of bacterial DNA was verified by PCR using the universal bacterial primers 27L (5′-AGAGTTTGATCATGGCTCAG-3′) and 1542R (5′-AAGGAGGTGATCCAGCCS-3′). Agarose gel analysis showed the absence of bands.

### 2.2. DNA Extraction and Libraries Preparation 

DNA was isolated using the standard phenol–chloroform method. The DNA concentration was measured using the Qubit 2.0 fluorimeter (Invitrogen, Carlsbad, CA, USA) according to the manufacturer’s instructions (extracted ~50 ng DNA). The extracted DNA was stored at −72 °C until further analysis. 

DNA was fragmented on Covaris S2 (Woburn, MA, USA) and libraries were pre-pared using the NEBNext Ultra II reagent kit (New England Biolabs, Ipswich, MA, USA). The resulting DNA libraries were sequenced on a Miseq instrument using the reagent kit v3 2×300 (Illumina, San Diego, CA, USA).

### 2.3. Bioinformatic Analysis 

An initial quality control was performed with the program Fast QC [36], then the data obtained were processed using Trimmomatic v. 0.36 [37], applying the parameter SLIDINGWINDOW:4:20, and sequences shorter than 50 nucleotides were removed from the analysis. A metagenomic “assembler” SPAdes v. 3.13.0 (Saint Petersburg, Russia) [38] was used to assemble de novo, mode metaspades, with default settings.

To identify open reading frames (ORFs), the collected contigs (≥500 nucleotides) from each virome were subjected to processing using Prodigal v. 2.6.3 with a parameter -meta [39]. Subsequently, each set was compared with the NCBI NR database (release 256, 2023) using the program Diamond v. 2.0.9 [40] with parameters -more-sensitive, -min-score 50, and e-value 10^−5^. Additionally, we used the database Integrated Microbial Genomes/Viral Resources v.4 (IMG/VR) [41].

To identify MCP virophages, we used hmmsearch from the HMMER 3.2.1 package [42] with 15 previously published models [20], e-value 10^−6^. For the analysis of complete MCP genes, the sequences with less than 500 amino acids were excluded from the analysis. According to the published data, the average MCP size is 593 amino acids ± 1 standard deviation (±40.1) [20]. Identical sequences from each sample are removed using Usearch v. 9.2.64 [43]. Identified proteins belonging to the major capsid protein were manually verified through online-blastp (NR). We also searched for closest relatives among the HQ-virophage MCP proteins [20] by performing a local blastp analysis with the parameter e-value 10^−5^.

For the phylogenetic tree based on the MCP proteins, the sequences were aligned us-ing the program MAFFT v. 7.407 [44] with the parameter -auto. The alignment was visually verified to remove partial and non-homologous sequences. TrimAl v. 1.2 (-gappyout) [45] was used to remove ambiguous regions. Trees were computed using IQ-TREE software v. 1.6.9 [46], model selection was performed using ModelFinder [47], and branch supports were determined using the approximate likelihood ratio test (1000 repetitions) [48] and the ultrafast bootstrap (1000 repetitions) [49]. The resulting trees were visualized and edited in iTOL [50].

The Unweighted Pair Group Method with Arithmetic Mean (UPGMA) is based on a distance matrix, obtained using the metric UniFrac (amino acid level) using the R programming language and packages phyloseq v. 1.38.0 [51], phangorn v. 2.11.1 [52], and vegan v. 2.6-2 [53], and the support nodes were calculated with pvclust v. 2.2-0 [54]. 

Contigs longer than 10,000 nucleotides from each sample were analyzed for the presence of the “core” genes of the virophages MCP, mCP, ATPase, and PRO, and affiliation was determined using an automatic classifier ICTV_VirophageSG (https://github.com/simroux/ICTV_VirophageSG, date of access 21 June 2023), and polinton-like viruses (PLVs) were also discovered. 

Virophage contigs having 4 “core” genes were dereplicated on the basis of 95% iden-tity over 80% of the length on the shortest contig applying the clustering scripts (CheckV v. 1.0.1) [55]. The presence of direct terminal repeats (DTRs) was determined using CheckV v. 1.0.1, and tRNAs were identified using the program tRNAscan-SE v. 2.0 [56]. The completeness of the genomes was assessed as in [21]: (1) by the presence of DTR, (2) by the length of linear genomes greater than 25 kbp, and (3) based on amino acid identity (AAI) predictions ≥90% (CheckV). Genome maps were visualized in SnapGene v. 6.0.2 (www.snapgene.com, date of access 4 April 2023). 

The mapping of reads to the genomes of virophages was performed in Bowtie 2 [57], followed by the use of Samtools v. 1.13 [58]. The samples were normalized to the lowest number of reads in the sample using the program SeqKit v. 2.3.0 [59].

## 3. Results

### 3.1. Taxonomy Viruses of Lake Baikal Viromes

According to the taxonomic analysis at the class level, bacteriophages of the class *Caudoviricetes* dominated in all samples (80–94.6%). The second most abundant class was *Megaviricetes*, which included the giant DNA viruses (with the exception of the BVP1 sample, where *Maveriviricetes* ranked second) and accounted for 5.6–17.9%. The third most abundant class was *Maveriviricetes*, which contained virophages (0.9–2.7%). 

**Figure 1:**
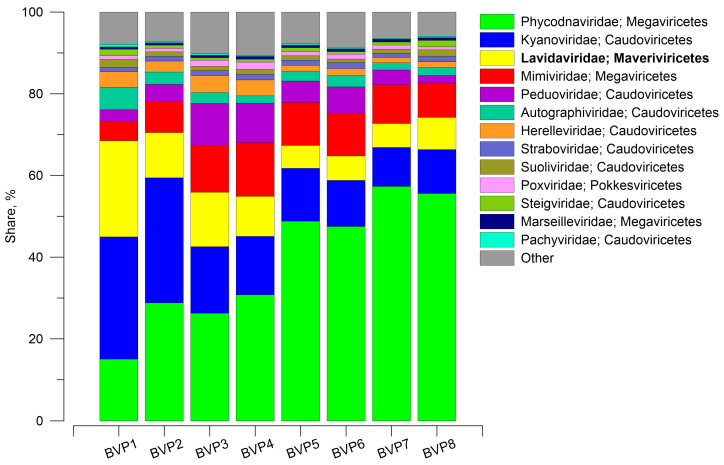
Taxonomic representation of viral ORFs at the level of families detected in Lake Baikal according to the NCBI NR database, blastp (e-value 10^−5^). Virophages are in bold. Others—less than 1%.

Analysis at the family level showed that *Kyanoviridae* (*Caudovoricetes*) was the most abundant in samples BVP1 and BVP2 (30% each), while in the remaining samples, the family *Phycodnaviridae* (*Megaviricetes*) was the most abundant (26.3 to 57.3%) (Figure 1). According to the currently accepted classification, the family of virophages *Lavidaviridae* (*Maveriviricetes*) accounted for 5 to 23.5% in our data. Thus, it was found that virophages occupy the top position in terms of representation.

### 3.2. Analyses of Virophages MCP Genes 

Using hmmsearch, 974 MCP-like sequences were identified. After the removal of short sequences (less than 500 aa), 319 (32.8%) amino acid sequences remained. The deletion of sequences that were not aligned and had no conserved regions resulted in 294 MCP proteins. The average length of the remaining sequences was 602 ± 36 (sd) amino acid residues, with a maximum length of 709 aa.

Their similarity to the proteins represented in the NCBI NR ranged from 23.8% to 90.1% (Table S1). The greatest similarity was observed with the Dishui Lake virophage (AMF83737), which had an aa similarity of 90.1%, with a coverage of 99.7% (sequence BVP7_NODE_1419_ORF7). The most highly represented close relatives were Dishui Lake virophage 2 (QIG59351), which corresponded to 24.8% of the estimated MCP from all samples with an aa similarity ranging from 36.7% to 80.4%, and Yellowstone Lake virophage 5 (YP_009177804)—20.1% of the sequences with a similarity ranging from 38.5% to 63.5%. 

We also compared the MCP proteins obtained in our study with those of HQ-virophages (Table S2). The greatest similarity was found for Ga0114980_10001820 (aa identity 96.2%, coverage 99%), which corresponded to the sequence BVP1_NODE_8945_ORF1. According to the description of this sequence [20], it was extracted from freshwater microbial communities from Lake Simoncouche (oligotrophic lake), Canada. The most abundant sequences were B570J40625_100003451 (aa identity 79.2–87.3%) and Ga0133913_10009135 (aa identity 48.5–51.8%), each corresponding to 8.2% of the Baikal MCP sequences. Virophage B570J40625_100003451 was extracted from freshwater microbial communities from Lake Mendota (eutrophic lake) and Ga0133913_10009135 was extracted from lakes in northern Canada (co-assembly). 

**Figure 2:**
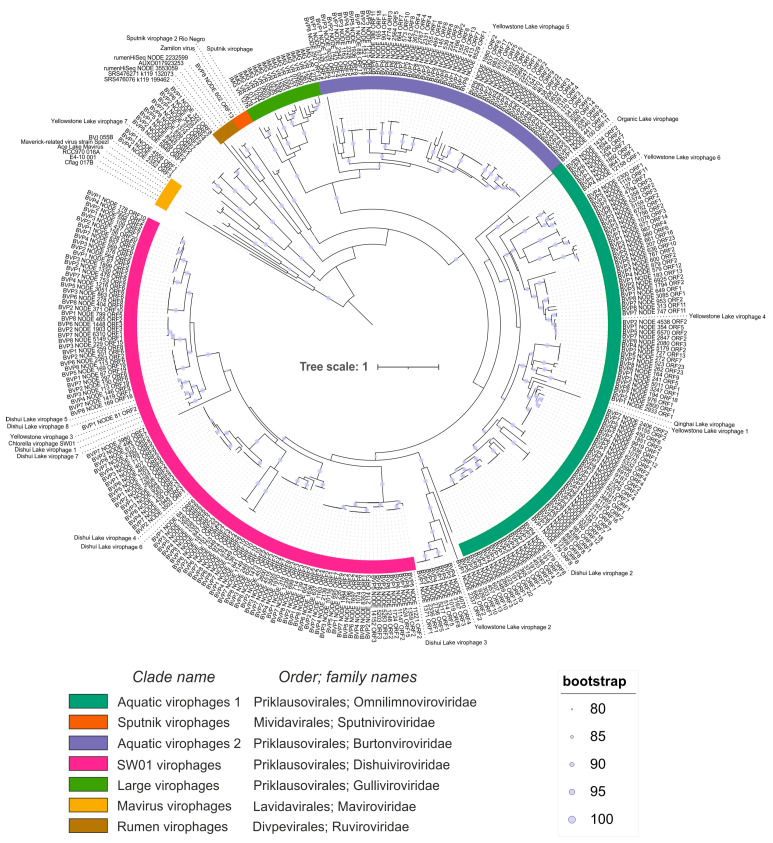
Virophage clades based on MCP phylogeny. Best-fit model according to BIC: LG + F + R8.

Phylogenetic analysis with known MCP proteins from the complete genomes of virophages showed their division into groups according to the new classification proposed by S. Roux et al. [21]. In total, we could identify seven groups with a support of more than 80% in the clade nodes calculated with two methods (Figure 2). The largest clades (containing the largest number of Baikal sequences) were SW01 virophages (38%)—named after the first isolated member, Aquatic virophages 1 (33.7%)—this group included representatives derived from a wide geographic range of freshwater lakes and Aquatic virophages 2 (20.1%)—most members of this cluster were related to large virophages.

One sequence (BVP8_NODE_602_ORF13) is located in the Sputnik virophages cluster and forms a separate branch. According to the blastp analysis, this sequence has the closest relative Sputnik virophage 2 (AUG85006, isolate Rio Negro), an aa identity of 27.2%, and a coverage of 92.6% (Table S1). It should be noted that the other two identified virophage proteins in the contig from which this MCP originated also show low similarity to known proteins—an aa identity of 23.9% with the hypothetical protein ASQ67_gp08 (YSLV 7), and an aa identity of 33.7% with the DNA packaging protein (Zamilon virus). 

No Baikal MCP sequences were included in the clades of Mavirus virophages, Large virophage, or Rumen virophages. Yellowstone Lake virophage 2 [21], which was not included in any group in the work of S. Roux et al., formed a joint cluster in the tree with 10 sequences from Lake Baikal from different samples (BVP1, BVP2, BVP3, BVP4, BVP5). The position of Yellowstone virophages 7, with which 13 sequences from Lake Baikal formed a common cluster, also remains unclear. In the original study, the MCP of YSLV7 is the most distant from other virophages in the phylogenetic analysis, suggesting a new lineage [14]. The taxonomic classification of virophages is very difficult, so further research is needed in this area, especially the analysis of genome structure.

**Figure 3:**
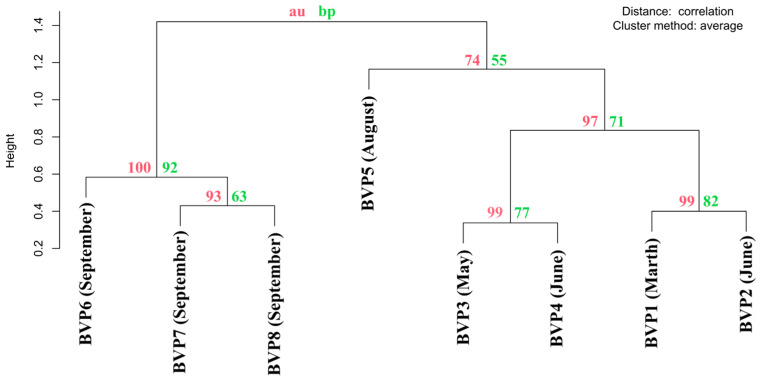
Cluster dendrogram with *p*-value (%) based on 294 MCP proteins. AU (Approximately Unbiased) *p*-value, BP (Bootstrap Probability) value.

Cluster analysis of the samples based on MCP phylogeny resulted in clustering by seasons. BVP1, BVP2, BVP3, and BVP4 were sampled in winter and spring. According to M. Kozhov [60], the beginning of June at Lake Baikal corresponds to biological spring, so we distinguished a strict “spring” cluster. BVP6, BVP7, and BVP8 formed an “autumn” (September) cluster, while BVP5 (August), which entered the clade with the spring samples, represented a separate branch, but the bootstrap support was relatively low (Figure 3). Thus, we can assume that the virophage community is seasonally influenced, which is apparently explained by pronounced seasonal fluctuations in the composition and structure of the planktonic community characteristic of Lake Baikal [61], including their hosts.

### 3.3. Identification of Complete or Nearly Complete Genomes of Virophages

In each set of contigs longer than 10,000 nucleotides, the automatic classifier ICTV_VirophageSG was able to identify between 5 and 32 contigs from each sample be-longing to putative virophages (Table 2); in addition, polinton-like viruses (PLVs) were identified (Table S3). Most of the putative virophage contigs belonged to the recently pro-posed families *Dishuiviroviridae* (42.5% virophage contigs) and *Omnilimnoviroviridae* (31.9%).

Of all 159 identified virophage sequences, only 7 had DTRs (i.e., circular complete genomes). Invert terminal repeats (ITR) were detected in two sequences identified as PLVs—BVP1_NODE_129 and BVP2_NODE_281. 

After the dereplication of virophage contigs (see Materials and Methods) having 4 “core” genes (65 contigs), 34 clusters (representative sequences) were obtained. Seventeen of the 34 contigs had AAI-based completeness >90%. Sequences that met the genome completeness criteria were assigned a Lake Baikal virophage (LBV) identifier (Table S4).

Transfer ribonucleic acid sequences (tRNAs) were encoded in LBV4 and LBV13 and recognized methionine (Met), anticodon CAT. We found no similarity between these sequences and known sequences in the NCBI NR or IMG/VR databases. 

**Figure 4:**
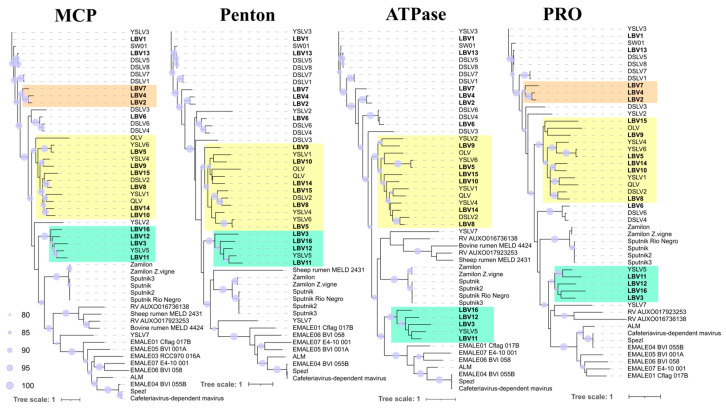
Unrooted maximum likelihood trees based on four core proteins. Supports in the nodes are indicated as over 80%. Sequences obtained from putative complete virophage genomes in this study are shown in bold. LBV—Lake Baikal virophage, DSLV—Dishui Lake virophage, YSLV—Yellowstone Lake virophage, OLV—Organic Lake virophage, RV—rumen virophage, ALM—Ace Lake Mavirus, Spezl—Maverick-related virus strain Spezl, QLM—Qinghai Lake virophage.

Trees with known virophages were constructed for all 4 genes from 16 contigs (Figure 4). In general, two clusters with highly supported Baikal sequences and their conservation for each protein were observed. In the MCP and PRO trees, three sequences form one cluster (LBV2, LBV4, LBV7), while in the Penton and ATPase trees, the LBV7 sequence is located in a neighboring branch. Two clusters included a great part of the sequences and formed a cluster with OLV, YSLV1, YSLV4, YSLV6, QLV, DSLV2 (six sequences) with YSLV5 (four sequences), i.e., according to the classification proposed by S. Roux, and they belonged to Aquatic virophage 1 and Aquatic virophage 2, respectively. 

**Figure 5:**
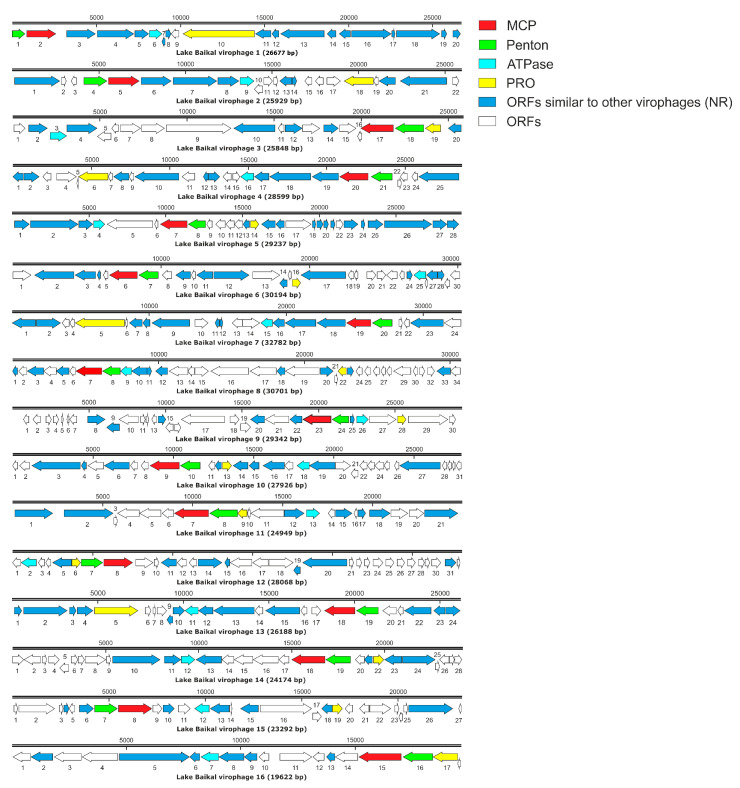
Genome maps of putative complete/near complete genomes of virophages identified in Lake Baikal viromes. Arrows indicate ORFs, direction indicates synthesis with + or—chains. The arrows below represent ORFs with overlapping reading frames.

A total of 16 putative complete genomes were obtained (Figure 5) that met the completeness criteria (longer than 25 kbp), and four “core” genes, completeness (CheckV) ≥ 90%). The number of ORFs in these contigs ranged from 18 to 34, and the GC-content was 26.6–45.2%. It is known that the genomes of virophages have a low GC-content [31]. LBV5, LBV7, LBV8, LBV14, and LBV15 had DTR. In addition to the identified four “core” genes, ORFs similar to eukaryote, bacteria, archaea, and viruses other than virophages were present in the genomes.

Among the identified hits of bacteria similar to the ORFs of virophages according to the NR database (total of 41 ORFs), 2 are represented as phage (tail fiber domain-containing protein, phage tail protein) and are, probably, prophages. At the same time, the remaining ORFs are similar to the bacterial proteins of the HNH endonuclease (WP_105774808), primosomal protein (WP_066855374), transcriptional regulator (MBT4479066), etc. A complete list can be found in Table S5. The amino acid similarity ranged from 25 to 73.9%. One ORF was similar to the archaeal sequence of a hypothetical protein (MCX6749161), with an aa identity of 72.4%. Five ORFs were similar to the eukaryote sequences (aa identity of 32.1–53.3%). In addition, 66.7% of all hits that were not from virophages belonged to hypothetical or uncharacterized proteins. The similarity to eukaryotes, bacteria, and archaea is probably due to the presence of metagenome-assembled genomes (MAG) in databases, which makes it difficult to determine the exact affiliation of the sequences.

Ten hits belonged to the phylum Nucleocytoviricota (representatives of giant viruses), among which *Phaeocystis globosa* virus, *Paramecium bursaria Chlorella* virus CVM-1, *Klosneuvirus KNV1*, and Organic Lake phycodnavirus 2 were identified. The amino acid similarity of these sequences ranged from 32.1 to 65.3%, probably, indicating horizontal gene transfer. 

Among the similar virophage ORFs, the integrase (CAI9421294), derived from the Maverick-related virus strain Spezl, was found only in the genome LBV9 (ORF_8), with an aa similarity of only 27.2%.

It should be noted that 34.5% of ORFs from 16 genomes had no significant hit, suggesting that virophages are underrepresented in the database. 

In addition, we note that MCP and penton located next to each other, which appears to be characteristic of most virophages, as previously mentioned [31]. Primase was present in all genomes (with the exception of LBV3, LBV5, LBV8, LBV9, LBV16), with the hit originating from the *Chlorella* virophage (ULY68422—represented as a supposed complex of primase–helicase), provirophage (Preplasmiviricota sp. Gezel-14T, YP_008059889), Yellowstone Lake virophage 5 (YP_009177814), Yellowstone Lake virophage 6 (YP_009177818), Qinghai Lake virophage (AIF72188), Dishui Lake virophage 4 (QIG59362), and Dishui Lake virophage 5 (QIG59403). At the same time, helicase (ARF12662) was revealed in LBV5, which is similar to *Klosneuvirus KNV1* (Nucleocytoviricota).

Analysis of the nucleotide similarity of the genomes to those available in the RefSeq database (release 218, blastn) showed that the closest sequences were Dishui Lake viropahge 8 (nucleotide identity—82.4%, coverage—90%) for LBV13 and Yellowstone Lake virophage 6 (nucleotide identity—87.0%, coverage—51%) for LBV5. 

**Figure 6:**
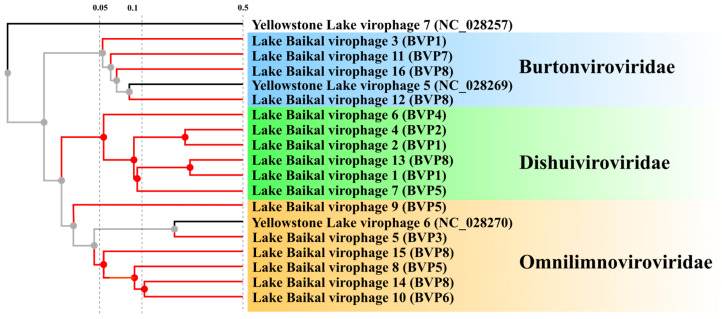
Proteomic tree constructed with the online service VipTree. Black branches show the closest relatives; red branches are from this study. For sequences from Lake Baikal, the sample to which the genome corresponds is given in parentheses; for the closest relatives, the accession number is given.

Three clusters are formed in the proteome tree, corresponding to three new families: *Burtonviroviridae*, *Dishuiviroviridae*, *Omnilimnoviroviridae*. As expected, YSLV7 forms a separate branch. Due to the limited number of sequences in the VipTree of virophages, only YSLV5, YSLV6, and YSLV7 are the closest relatives (Figure 6). 

**Figure 7:**
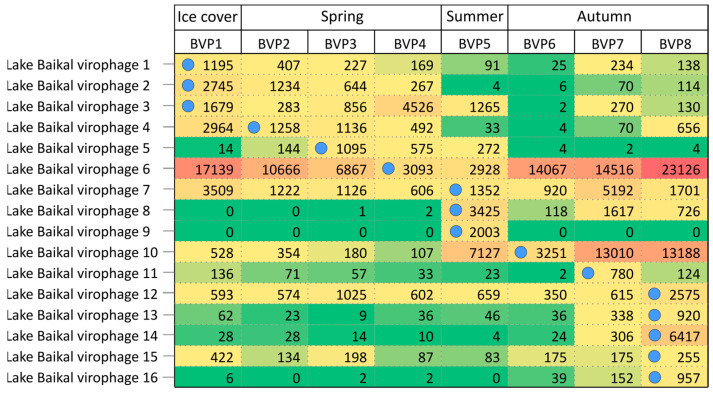
Mapping of reads to 16 putative virophage genomes. The number of aligned reads was normalized. Value—the number of reads. The blue circles show from the reads of which sample the genome was assembled.

The mapping of reads on 16 Lake Baikal virophage genomes showed that 4 of them were in all seasons (LBV6, LBV7, LBV10, LBV12) with a number of reads of more than 100 (Figure 7). LBV6 was most strongly represented in all samples. LBV9 was only characteristic of the BVP5 sample (Maloye More Strait). LBV8 and LBV10 were more prevalent in the summer and autumn samples (BVP5, BVP6, BVP7, BVP8). LBV14 had the greater part of reads only in the sample from which it was extracted (BVP8). LBV16 was identified in BVP7 and BVP8, and the lowest number of reads were detected in other seasons, as well as LBV13 and LBV14. 

## 4. Discussion

Virophages are currently a little-studied element in the viral community, but in recent years, the number of studies focusing on this topic has increased.

Here, for the first time we successfully detailed searched for and identified virophages in the metagenomes of the viral fraction from Lake Baikal. Virophages were found to be present in all seasons, and they were not confined to a greater extent to either the deep-water pelagic basins or the shallow water strait. Based on the MCP cluster analysis, it was shown that virophages from different seasons form their own clusters. Thus, we can assume that the virophage community is subject to seasonal influences, which could be obviously explained by pronounced seasonal fluctuations in the composition and structure of the planktonic community characteristic of Lake Baikal [61], including their hosts.

According to the newly proposed classification of virophages, we performed phylo-genetic analysis of all the obtained MCPs and showed that clustering by groups generally reveals a clear distribution pattern. In the Baikal samples, the sequences of four groups were identified and no sequences belonging to the groups Mavirus virophages, Large virophages, or Rumen virophages by S. Roux et al. [21] were found. At the same time, we observed an expansion of the clusters with YSLV2 and YSLV7, which obviously represent new lineages. 

The trees constructed on the basis of four conserved proteins (MCP, penton, ATPase, and PRO) showed three groups, conserved for all four proteins (except for certain sequences).

By applying the automatic classifier and genome completeness criteria, we managed to identify 16 putative complete genomes of virophages with 4 conserved proteins. Some virophage genomes, such as those collected from the sheep rumen metagenome, appeared to lack the penton gene [15], but as previously mentioned, we had found no similarities with members of this clade.

Some genes found in the virophage genomes showed diverse similarities with giant viruses, phages, bacteria, eukaryote, and mobile genetic elements [31]. It has been repeatedly shown that some virophage genes have similarities with genes in other viruses (giant viruses, bacteriophages) and bacteria, for example, in [1,10,11]. We have also observed such similarities with non-virophage sequences in our genomes. It has previously been suggested that the common gene of integrase for Sputnik and archaeal viruses (plasmids) could have been isolated independently from an ancestral virus or may reside in an archaeal endosymbiont located in a eukaryotic cell [1]. Mavirus, for example, has a close relationship with the Maverick/Polinton virus-like mobile elements [2], which in turn are found in a wide range of eukaryotes [62]. A noteworthy detail is that polintons most likely originated from bacteriophages and give rise to the evolution of most major eukaryotic dsDNA viruses, as well as several groups of plasmids and transposons [63]. Moreover, polinton-like virus Gezel-14T was most recently shown to be capable of forming virions [64]. In our opinion, the presence of similar sequences in giant viruses and eukaryotic hosts naturally reflects their close relationship and the process of horizontal gene transfer. On the other hand, the similarities with eukaryotes, bacteria, and archaea may be due to the MAGs present in the databases, due to which sequences may be incorrectly determined.

The prediction of the host virus for virophages is difficult, which is also due to the small number of cultured virophages. The study by S. Roux [16], for example, used a set of co-occurrence analyses, but the authors warned that the results of this analysis should be interpreted with caution. In our dataset, we identified potential hosts based only on sequence similarity with known Nucleocytoviricota, i.e., potential hosts of virophages, but this only showed a possible range of hosts. As for eukaryotic hosts, it is difficult to draw conclusions as we are using a fraction smaller than 0.2 μm and the main pool of eukaryotic DNA is truncated during filtration by the removal of phyto- and zooplankton. However, in all samples, blastp analysis of all ORFs by the RefSeq database reveals a few ORFs similar to representatives such as picophytoplankton green flagellates *Micromonas commoda* (aa similarity of 23.7–95.2%) and *Ostreococcus lucimarinus* CCE9901 (aa similarity of 21.9–93.3%). Despite the fact that the above-mentioned representatives are marine species, there are, probably, close species in Lake Baikal. Nowadays, the prasinophytes of Lake Baikal have not been described according to morphological criteria, but using high-throughput sequencing of 18S rRNA, and amplicon sequences belonging to the family *Mamiellophyceae* (*Prasinophyceae*, *Chlorophyta*) are found in plankton [65]. Only in the PLV sequences ORF similar to Fusarium oxysporum Fo47 (*Ascomycota*) was detected, and the remaining sequences obtained after ICTV_VirophageSG did not contain any ORF similar to the eukaryotic sequence.

tRNA genes have been predicted in the viruses of ssDNA (single-stranded DNA), ssRNA (single-stranded RNA) viruses, and many other viruses with dsDNA [66,67]. The presence of the tRNA genes in the genomes of viruses should compensate for differences in codon and/or amino acid usage between the virus and host, thus promoting efficient protein synthesis and/or thereby expanding the host range [67,68]. The origin of these tRNAs in virophages remains unclear. Previously, tRNA was identified in HQ-virophages, and seven genomes of them contained the integrase gene, supporting the hypothesis that it was possible to integrate into the host genome [20]. In our data, we did not identify integrase in genomes containing tRNA (LBV4 and LBV13). Therefore, the function of tRNA in virophages needs to be unraveled in further studies.

The presence of most genotypes in different basins and seasons indicates that virophages are widely distributed at Lake Baikal in all seasons. The great similarity of the sequences we obtained with those of the virophages of Lake Dishui (China), Yellowstone Lake (USA), Ga0114980_10001820 (Lake Simoncouche, Canada), and B570J40625_100003451 (Lake Mendota, USA) proves their global distribution. 

Our data expand knowledge of virophages both in general and in freshwater lakes in particular, especially in lakes of ancient origin.

## 5. Conclusions

Virophages are still a relatively understudied subject that represent a unique group of viruses. Our data, obtained from the deepest and oldest freshwater lake on the planet, will contribute to the understanding of the distribution, genetic composition, and host relationships of these viruses. By analyzing eight metagenomes of the viral fraction (smaller than 0.2 μm) obtained in different seasons, as well as from different basins and straits, we were able to detect their presence in all seasons. Determining the taxonomic affiliation of new viruses is a difficult task. The identification of virophages in different habitats and the formation of a data pool should eventually clarify their genetic diversity and possibly reveal patterns in the composition of virophage communities. In addition, we identified 294 MCP genes that potentially extend new lineages, as shown for YSLV2 and YSLV7 in phylogenetic trees. 

## Figures and Tables

**Table 1:** Dates and coordinates of the specimens sampling from Lake Baikal.

Sample	Data	Latitude/Longitude
BVP1	22.03.2018	51.798082 N, 104.876782 E
BVP2	8.06.2018	51.820000 N, 104.900000 E
BVP3	31.05.2018	52.990000 N, 108.170000 E
BVP4	4.06.2018	54.550000 N, 108.710000 E
BVP5	5.08.2018	53.283333 N, 107.350000 E
BVP6	27.09.2018	51.721883 N, 104.993283 E
BVP7	25.09.2018	53.011111 N, 107.730000 E
BVP8	23.09.2018	54.450000 N, 109.139722 E

**Table 2:** Number of detected contigs of putative virophages and PLVs in samples.

Sample	Number of Sequences of Virophages	Number of Contigs Containing ATPase, MCP, PRO, Penton	Length of Contigs Containing ATPase, MCP, PRO, Penton (kbp)	Number of PLVs Sequences
BVP1	32	14	12–29.5	21
BVP2	19	7	11.8–31	17
BVP3	17	7	13.4–31.7	5
BVP4	9	3	18.3–31.2	1
BVP5	20	10	10.8–32.8	10
BVP6	5	3	20.4–27.9	6
BVP7	31	11	10–30.9	3
BVP8	26	10	10.3–31.5	4
Total	159	65	-	67

## Data Availability

Raw fastq files were deposited into the Sequence Read Archive (SRA) NCBI under the project number PRJNA1006167. Fasta files with MCP sequences can be found at https://github.com/SergeyBaikal/Virophages-found-in-viromes-from-Lake-Baikal (accessed on 5 December 2023). The putative complete genomes of virophages from Lake Baikal have been deposited in Genbank under numbers OR738444-OR738462.

## Supplementary Materials

The following supporting information can be downloaded at: https://www.mdpi.com/article/10.3390/biom13121773/s1, Table S1: Closest relatives identified through comparison 294 MCPs with the NCBI NR database; Table S2: Results obtained by comparison MCPs obtained in our study with those of HQ-virophages; Table S3: The result of contig analysis for the presence MCPs, pentons, ATPases, PROs, PLVs using automatic classifier ICTV_VirophageSG; Table S4: Metadata of identified 16 putative virophage genomes; Table S5: Hits identified through comparison ORFs from putative complete genomes with the NR database.

## References

[1] La Scola B., Desnues C., Pagnier I., Robert C., Barrassi L., Fournous G., Merchat M., Suzan-Monti M., Forterre P., Koonin E. (2008). The Virophage as a Unique Parasite of the Giant Mimivirus. Nature.

[2] Fischer M.G., Suttle C.A. (2011). A Virophage at the Origin of Large DNA Transposons. Science.

[3] Campos R.K., Boratto P.V., Assis F.L., Aguiar E.R., Silva L.C., Albarnaz J.D., Dornas F.P., Trindade G.S., Ferreira P.P., Marques J.T. (2014). Samba Virus: A Novel Mimivirus from a Giant Rain Forest, the Brazilian Amazon. Virol. J..

[4] Desnues C., La Scola B., Yutin N., Fournous G., Robert C., Azza S., Jardot P., Monteil S., Campocasso A., Koonin E.V. (2012). Provirophages and Transpovirons as the Diverse Mobilome of Giant Viruses. Proc. Natl. Acad. Sci. USA.

[5] Gaia M., Pagnier I., Campocasso A., Fournous G., Raoult D., La Scola B. (2013). Broad Spectrum of Mimiviridae Virophage Allows Its Isolation Using a Mimivirus Reporter. PLoS ONE.

[6] Gaia M., Benamar S., Boughalmi M., Pagnier I., Croce O., Colson P., Raoult D., La Scola B. (2014). Zamilon, a Novel Virophage with Mimiviridae Host Specificity. PLoS ONE.

[7] Bekliz M., Verneau J., Benamar S., Raoult D., La Scola B., Colson P. (2015). A New Zamilon-like Virophage Partial Genome Assembled from a Bioreactor Metagenome. Front. Microbiol..

[8] Stough J.M.A., Yutin N., Chaban Y.V., Moniruzzaman M., Gann E.R., Pound H.L., Steffen M.M., Black J.N., Koonin E.V., Wilhelm S.W. (2019). Genome and Environmental Activity of a Chrysochromulina Parva Virus and Its Virophages. Front. Microbiol..

[9] Azevedo B.L.D., Júnior J.P.A., Ullmann L.S., Rodrigues R.A.L., Abrahão J.S. (2022). The Discovery of a New Mimivirus Isolate in Association with Virophage-Transpoviron Elements in Brazil Highlights the Main Genomic and Evolutionary Features of This Tripartite System. Viruses.

[10] Yau S., Lauro F.M., DeMaere M.Z., Brown M.V., Thomas T., Raftery M.J., Andrews-Pfannkoch C., Lewis M., Hoffman J.M., Gibson J.A. (2011). Virophage Control of Antarctic Algal Host–Virus Dynamics. Proc. Natl. Acad. Sci. USA.

[11] Zhou J., Zhang W., Yan S., Xiao J., Zhang Y., Li B., Pan Y., Wang Y. (2013). Diversity of Virophages in Metagenomic Data Sets. J. Virol..

[12] Gong C., Zhang W., Zhou X., Wang H., Sun G., Xiao J., Pan Y., Yan S., Wang Y. (2016). Novel Virophages Discovered in a Freshwater Lake in China. Front. Microbiol..

[13] Oh S., Yoo D., Liu W.-T. (2016). Metagenomics Reveals a Novel Virophage Population in a Tibetan Mountain Lake. Microbes Environ..

[14] Zhou J., Sun D., Childers A., McDermott T.R., Wang Y., Liles M.R. (2015). Three Novel Virophage Genomes Discovered from Yellowstone Lake Metagenomes. J. Virol..

[15] Yutin N., Kapitonov V.V., Koonin E. (2015). V A New Family of Hybrid Virophages from an Animal Gut Metagenome. Biol. Direct.

[16] Roux S., Chan L.-K., Egan R., Malmstrom R.R., McMahon K.D., Sullivan M.B. (2017). Ecogenomics of Virophages and Their Giant Virus Hosts Assessed through Time Series Metagenomics. Nat. Commun..

[17] Sheng Y., Wu Z., Xu S., Wang Y. (2022). Isolation and Identification of a Large Green Alga Virus (Chlorella Virus XW01) of Mimiviridae and Its Virophage (Chlorella Virus Virophage SW01) by Using Unicellular Green Algal Cultures. J. Virol..

[18] Tokarz-Deptuła B., Chrzanowska S., Gurgacz N., Stosik M., Deptuła W. (2023). Virophages—Known and Unknown Facts. Viruses.

[19] Krupovic M., Kuhn J.H., Fischer M.G. (2016). A Classification System for Virophages and Satellite Viruses. Arch. Virol..

[20] Paez-Espino D., Zhou J., Roux S., Nayfach S., Pavlopoulos G.A., Schulz F., McMahon K.D., Walsh D., Woyke T., Ivanova N.N. (2019). Diversity, Evolution, and Classification of Virophages Uncovered through Global Metagenomics. Microbiome.

[21] Roux S., Fischer M.G., Hackl T., Katz L.A., Schulz F., Yutin N. (2023). Updated Virophage Taxonomy and Distinction from Polinton-like Viruses. Biomolecules.

[22] Sobhy H. (2018). Virophages and Their Interactions with Giant Viruses and Host Cells. Proteomes.

[23] Pagnier I., Reteno D.-G.I., Saadi H., Boughalmi M., Gaia M., Slimani M., Ngounga T., Bekliz M., Colson P., Raoult D. (2013). A Decade of Improvements in Mimiviridae and Marseilleviridae Isolation from Amoeba. Intervirology.

[24] Yoosuf N., Yutin N., Colson P., Shabalina S.A., Pagnier I., Robert C., Azza S., Klose T., Wong J., Rossmann M.G. (2012). Related Giant Viruses in Distant Locations and Different Habitats: Acanthamoeba Polyphaga Moumouvirus Represents a Third Lineage of the Mimiviridae That Is Close to the Megavirus Lineage. Genome Biol. Evol..

[25] Arslan D., Legendre M., Seltzer V., Abergel C., Claverie J.-M. (2011). Distant Mimivirus Relative with a Larger Genome Highlights the Fundamental Features of Megaviridae. Proc. Natl. Acad. Sci. USA.

[26] Santini S., Jeudy S., Bartoli J., Poirot O., Lescot M., Abergel C., Barbe V., Wommack K.E., Noordeloos A.A.M., Brussaard C.P.D. (2013). Genome of Phaeocystis Globosa Virus PgV-16T Highlights the Common Ancestry of the Largest Known DNA Viruses Infecting Eukaryotes. Proc. Natl. Acad. Sci. USA.

[27] Blanc G., Gallot-Lavallée L., Maumus F. (2015). Provirophages in the Bigelowiella Genome Bear Testimony to Past Encounters with Giant Viruses. Proc. Natl. Acad. Sci. USA.

[28] Hackl T., Duponchel S., Barenhoff K., Weinmann A., Fischer M.G. (2021). Virophages and Retrotransposons Colonize the Genomes of a Heterotrophic Flagellate. eLife.

[29] Mougari S., Sahmi-Bounsiar D., Levasseur A., Colson P., La Scola B. (2019). Virophages of Giant Viruses: An Update at Eleven. Viruses.

[30] Bekliz M., Colson P., La Scola B. (2016). The Expanding Family of Virophages. Viruses.

[31] Fischer M.G. (2021). The Virophage Family Lavidaviridae. Curr. Issues Mol. Biol..

[32] Potapov S.A., Tikhonova I.V., Krasnopeev A.Y., Kabilov M.R., Tupikin A.E., Chebunina N.S., Zhuchenko N.A., Belykh O.I. (2019). Metagenomic Analysis of Virioplankton from the Pelagic Zone of Lake Baikal. Viruses.

[33] Potapov S., Gorshkova A., Krasnopeev A., Podlesnaya G., Tikhonova I., Suslova M., Kwon D., Patrushev M., Drucker V., Belykh O. (2023). RNA-Seq Virus Fraction in Lake Baikal and Treated Wastewaters. Int. J. Mol. Sci..

[34] Potapov S., Belykh O., Krasnopeev A., Gladkikh A., Kabilov M., Tupikin A., Butina T. (2018). Assessing the Diversity of the *G23* Gene of T4-like Bacteriophages from Lake Baikal with High-Throughput Sequencing. FEMS Microbiol. Lett..

[35] Butina T.V., Bukin Y.S., Petrushin I.S., Tupikin A.E., Kabilov M.R., Belikov S.I. (2021). Extended Evaluation of Viral Diversity in Lake Baikal through Metagenomics. Microorganisms.

[36] Andrews S. FastQC: A Quality Control Tool for High Throughput Sequence Data. http://www.bioinformatics.babraham.ac.uk/projects/fastqc/.

[37] Bolger A.M., Lohse M., Usadel B. (2014). Trimmomatic: A Flexible Trimmer for Illumina Sequence Data. Bioinformatics.

[38] Bankevich A., Nurk S., Antipov D., Gurevich A.A., Dvorkin M., Kulikov A.S., Lesin V.M., Nikolenko S.I., Pham S., Prjibelski A.D. (2012). SPAdes: A New Genome Assembly Algorithm and Its Applications to Single-Cell Sequencing. J. Comput. Biol..

[39] Hyatt D., Chen G.-L., LoCascio P.F., Land M.L., Larimer F.W., Hauser L.J. (2010). Prodigal: Prokaryotic Gene Recognition and Translation Initiation Site Identification. BMC Bioinform..

[40] Buchfink B., Xie C., Huson D.H. (2014). Fast and Sensitive Protein Alignment Using DIAMOND. Nat. Methods.

[41] Camargo A.P., Nayfach S., Chen I.-M.A., Palaniappan K., Ratner A., Chu K., Ritter S.J., Reddy T.B.K., Mukherjee S., Schulz F. (2023). IMG/VR v4: An Expanded Database of Uncultivated Virus Genomes within a Framework of Extensive Functional, Taxonomic, and Ecological Metadata. Nucleic Acids Res..

[42] Eddy S.R. (2011). Accelerated Profile HMM Searches. PLoS Comput. Biol..

[43] Edgar R.C. (2010). Search and Clustering Orders of Magnitude Faster than BLAST. Bioinformatics.

[44] Katoh K., Standley D.M. (2013). MAFFT Multiple Sequence Alignment Software Version 7: Improvements in Performance and Usability. Mol. Biol. Evol..

[45] Capella-Gutiérrez S., Silla-Martínez J.M., Gabaldón T. (2009). TrimAl: A Tool for Automated Alignment Trimming in Large-Scale Phylogenetic Analyses. Bioinformatics.

[46] Nguyen L.-T., Schmidt H.A., von Haeseler A., Minh B.Q. (2015). IQ-TREE: A Fast and Effective Stochastic Algorithm for Estimating Maximum-Likelihood Phylogenies. Mol. Biol. Evol..

[47] Kalyaanamoorthy S., Minh B.Q., Wong T.K.F., von Haeseler A., Jermiin L.S. (2017). ModelFinder: Fast Model Selection for Accurate Phylogenetic Estimates. Nat. Methods.

[48] Guindon S., Dufayard J.-F., Lefort V., Anisimova M., Hordijk W., Gascuel O. (2010). New Algorithms and Methods to Estimate Maximum-Likelihood Phylogenies: Assessing the Performance of PhyML 3.0. Syst. Biol..

[49] Hoang D.T., Chernomor O., von Haeseler A., Minh B.Q., Vinh L.S. (2018). UFBoot2: Improving the Ultrafast Bootstrap Approximation. Mol. Biol. Evol..

[50] Letunic I., Bork P. (2007). Interactive Tree Of Life (ITOL): An Online Tool for Phylogenetic Tree Display and Annotation. Bioinformatics.

[51] McMurdie P.J., Holmes S. (2013). Phyloseq: An R Package for Reproducible Interactive Analysis and Graphics of Microbiome Census Data. PLoS ONE.

[52] Schliep K.P. (2011). Phangorn: Phylogenetic Analysis in R. Bioinformatics.

[53] Oksanen J., Simpson G.L., Blanchet F.G., Kindt R., Legendre P., Minchin P.R., O’Hara R.B., Solymos P. Vegan: Community Ecology Package. https://cran.r-project.org/web/packages/vegan/index.html.

[54] Suzuki R., Terada Y., Shimodaira H. Hierarchical Clustering with P-Values via Multiscale Bootstrap Resampling. https://github.com/shimo-lab/pvclust.

[55] Nayfach S., Camargo A.P., Schulz F., Eloe-Fadrosh E., Roux S., Kyrpides N.C. (2021). CheckV Assesses the Quality and Completeness of Metagenome-Assembled Viral Genomes. Nat. Biotechnol..

[56] Chan P.P., Lin B.Y., Mak A.J., Lowe T.M. (2021). TRNAscan-SE 2.0: Improved Detection and Functional Classification of Transfer RNA Genes. Nucleic Acids Res..

[57] Langmead B., Salzberg S.L. (2012). Fast Gapped-Read Alignment with Bowtie 2. Nat. Methods.

[58] Li H., Handsaker B., Wysoker A., Fennell T., Ruan J., Homer N., Marth G., Abecasis G., Durbin R. (2009). The Sequence Alignment/Map Format and SAMtools. Bioinformatics.

[59] Shen W., Le S., Li Y., Hu F. (2016). SeqKit: A Cross-Platform and Ultrafast Toolkit for FASTA/Q File Manipulation. PLoS ONE.

[60] Kozhov M.M. (1962). Biologiya Ozera Baikal (Biology of Lake Baikal).

[61] Kozhova O.M., Izmest’eva L.R. (1998). Lake Baikal: Evolutionand Biodiversity.

[62] Kapitonov V.V., Jurka J. (2006). Self-Synthesizing DNA Transposons in Eukaryotes. Proc. Natl. Acad. Sci. USA.

[63] Krupovic M., Koonin E.V. (2015). Polintons: A Hotbed of Eukaryotic Virus, Transposon and Plasmid Evolution. Nat. Rev. Microbiol..

[64] Roitman S., Rozenberg A., Lavy T., Brussaard C.P.D., Kleifeld O., Béjà O. (2023). Isolation and Infection Cycle of a Polinton-like Virus Virophage in an Abundant Marine Alga. Nat. Microbiol..

[65] Annenkova N.V., Giner C.R., Logares R. (2020). Tracing the Origin of Planktonic Protists in an Ancient Lake. Microorganisms.

[66] de Paula Oliveira H., dos Santos E.R., Harrison R.L., Ribeiro B.M., Ardisson-Araújo D.M.P. (2022). Identification and Analysis of Putative TRNA Genes in Baculovirus Genomes. Virus Res..

[67] Morgado S., Vicente A. (2019). Global In-Silico Scenario of TRNA Genes and Their Organization in Virus Genomes. Viruses.

[68] Bailly-Bechet M., Vergassola M., Rocha E. (2007). Causes for the Intriguing Presence of TRNAs in Phages. Genome Res..

